# A local operational definition of pediatric emergency department overcrowding: an 8-year retrospective observational cohort study from the OACS+ project

**DOI:** 10.3389/fped.2026.1882596

**Published:** 2026-08-05

**Authors:** Johanna T. Meßner, Dennis Freuer, Christine Meisinger, Birgit Prodinger, Bin Zhou, Melanie L. Conrad, Anne Ch. Garbe, Anna-Lena Behr, Anna Juraschek, Fabian B. Fahlbusch, Florian Weber

**Affiliations:** 1 Interdisciplinary Pediatric Emergency Department, Faculty of Medicine, University of Augsburg, Augsburg, Germany; 2 Epidemiology, Faculty of Medicine, University of Augsburg, University Hospital Augsburg, Augsburg, Germany; 3 Chair of Inclusive Health Care, Faculty of Medicine, University of Augsburg, Augsburg, Germany; 4 Chair of Model-based Environmental Exposure Science, Environmental Health Sciences Institute, Faculty of Medicine, University of Augsburg, Augsburg, Germany; 5 Neonatology and Pediatric Intensive Care, Faculty of Medicine, University of Augsburg, Augsburg, Germany; 6 Bayerisches Zentrum für präventive Infektionsmedizin (BZI), Department of Pediatrics and Adolescent Medicine, Medical Faculty, University of Augsburg, Augsburg, Germany

**Keywords:** LOS, LWBS, overcrowding, pediatric emergency care, pediatric emergency department

## Abstract

**Background:**

Emergency department (ED) overcrowding is increasingly observed worldwide and is associated with impaired care quality and patient outcomes. However, no universally accepted definition or measurement method exists. This challenge is particularly relevant in pediatric EDs (PEDs), where crowding is shaped by patient volume, staffing, local care structures, spatial capacity, and temporally fluctuating demand, including infectious surges.

**Objective:**

This study aimed to derive a locally applicable operational definition of PED overcrowding reflecting the care structures and demand patterns of the University Hospital Augsburg (UKA) PED and supporting future validation, prediction, and intervention studies.

**Methods:**

We conducted an 8-year retrospective cohort study (2017–2024) including 141,008 PED visits by children aged 0–18 years at UKA. Candidate parameters included patients leaving without being seen (LWBS), length of stay (LOS), time to triage, and patient-to-staff ratio (ps-ratio). Descriptive analyses covered 2017–2024, whereas ps-ratio analyses were restricted to 2020–2024, when nursing staffing data were available. Correlation analyses assessed bivariate relationships. ROC analyses derived local ps-ratio thresholds using daily LWBS events, operationalized as ≥1 and ≥2 events/day, as binary internal reference anchors. Time-series models examined temporal trends and lockdown-related shifts.

**Results:**

The ps-ratio showed the strongest correlations with LOS (*r* = 0.612) and LWBS (*r* = 0.428). ROC analyses identified local ps-ratio thresholds between 2.03 and 2.20 patients per nurse, depending on the LWBS anchor and model specification. A rounded threshold of approximately 2.2 patients per nurse showed moderate and broadly stable discrimination (raw ≥ 1 LWBS/day: AUC = 0.73). Time-series modeling for 2020–2024 showed a nonlinear ps-ratio trend and a marked level shift during the COVID-19 lockdown period. PED demand was characterized by high self-referral rates, predominantly lower-acuity presentations, weekend and holiday clustering, and temporal fluctuations in visit volume.

**Conclusions:**

Locally derived operational definitions may improve interpretation of PED overcrowding by linking patient demand to staffing capacity and local care structures. In the OACS + cohort, the ps-ratio emerged as a staffing-sensitive, clinically interpretable operational marker. The proposed threshold should be considered a locally derived exploratory benchmark requiring external validation before application elsewhere.

## 1 Introduction

Crowding of healthcare systems and medical facilities is a long-standing problem that has intensified in recent years (1, 2). Both adult and pediatric Emergency Departments (EDs) are experiencing steadily increasing numbers of patients seeking emergency care worldwide (3–5). When the need for emergency care exceeds an ED's available physical space, staffing, and equipment, timely and high-quality care becomes difficult to maintain. In this context, overcrowding is commonly described as a mismatch between the care needs of patients and the resources an ED can provide, resulting in prolonged waiting times, delayed boarding and treatment, as well as impaired throughput (1, 6, 7). The consequences are substantial, including diminished quality of care, higher in-hospital mortality and morbidity, increased diagnostic and therapeutic errors, and reduced satisfaction among patients and staff (5, 8–13). While numerous solutions and mitigation strategies have been proposed (5, 7, 8), sustainable interventions depend on a clear and operationally meaningful definition of overcrowding and its drivers.

A universally accepted metric that defines when an ED becomes overcrowded is still lacking for both adult and pediatric settings (14–16). Existing definitions often rely on outcome-oriented indicators—such as the number of patients leaving without being seen (LWBS), ED length of stay (LOS), or occupancy—or on multidimensional scores including the National Emergency Department Overcrowding Scale (NEDOCS) and the Emergency Department Work Index (EDWIN) (3, 6, 17). These measures were largely developed and validated in adult EDs, but evidence specifically testing their performance in settings providing emergency care for children remains scarce (18, 19). Pediatric-specific tools have been introduced, including the Pediatric Emergency Department Overcrowding Score (PEDOCS) (20) and the SOTU-PED (Score Objectif de Tension dans les services d'Urgences pédiatriques) (21), yet no gold standard has emerged. A central reason for this may be that overcrowding is not a purely abstract or universally uniform state, but a context-dependent systems phenomenon. In PEDs in particular, crowding is shaped by the interaction between the care needs of children and local structures of care, including staffing models, triage procedures, room availability, hospital throughput, and access to outpatient alternatives. In addition, the number of children requiring emergency care is highly dynamic and can be substantially modified by seasonal infectious waves and changing care-seeking behavior.

ED overcrowding is commonly conceptualized as the result of interacting input, throughput, and output factors, including low-acuity presentations, staffing and diagnostic bottlenecks, treatment-space limitations, boarding, and access block. Therefore, any operational metric should be interpreted in relation to the local patient-flow structure, available resources, and downstream inpatient capacity (22, 23).

This multidimensional framework is particularly relevant when selecting measurable indicators of crowding. Many current metrics primarily quantify consequences of crowding, such as delayed care, LWBS, or prolonged LOS. They may less directly capture upstream determinants that are particularly relevant in pediatrics, including infectious surges, variation in case mix, staffing constraints, and spatial capacity. Consequently, the same numerical value may reflect different operational realities across PEDs, and a marker that performs well in one setting may be less informative in another if the local care environment is not considered (18, 23).

Against this background, the primary objective of this study was to derive a locally applicable operational definition of overcrowding for the University Hospital Augsburg PED. Longitudinal routine data from an 8-year period were used to describe children and adolescents treated in the PED and to characterize temporal patterns in volume and acuity. Guided by the current literature and the descriptive analyses, candidate overcrowding markers were evaluated for plausibility, internal coherence, and correlation structure. The overarching aim was to establish a context-grounded framework for defining local overcrowding that can support future validation, predictive modeling, and intervention studies.

## 2 Methods

### 2.1 Study design and setting

This retrospective observational study was conducted at the tertiary pediatric emergency department (PED) of University Hospital Augsburg (Universitätsklinikum Augsburg; UKA), Bavaria, Germany, within the OACS + Project (Overcrowding—Analysis, Concepts and Strategies), an interdisciplinary pediatric emergency care and epidemiology initiative focused on operational crowding metrics and temporal patterns in emergency care demand among children and adolescents in pediatric emergency departments. Reporting was guided by the STROBE recommendations for observational studies. UKA represents a maximum-care university hospital within the regional health care network of Bavaria. Under the German Federal Joint Committee (G-BA) framework, it is classified as a level 3 emergency care hospital, fulfilling the criteria for comprehensive emergency services and operating a dedicated pediatric emergency care module (24, 25). G-BA is the highest decision-making body of the joint self-government of physicians, hospitals, and statutory health insurance funds in Germany.

Structurally, the PED includes 4 fully equipped treatment rooms with central monitoring, with the option of adding one additional treatment room after 3:30 p.m. and on weekends. The waiting area accommodates approximately 20 children and adolescents, depending on the number of accompanying family members.

**Figure 1:**
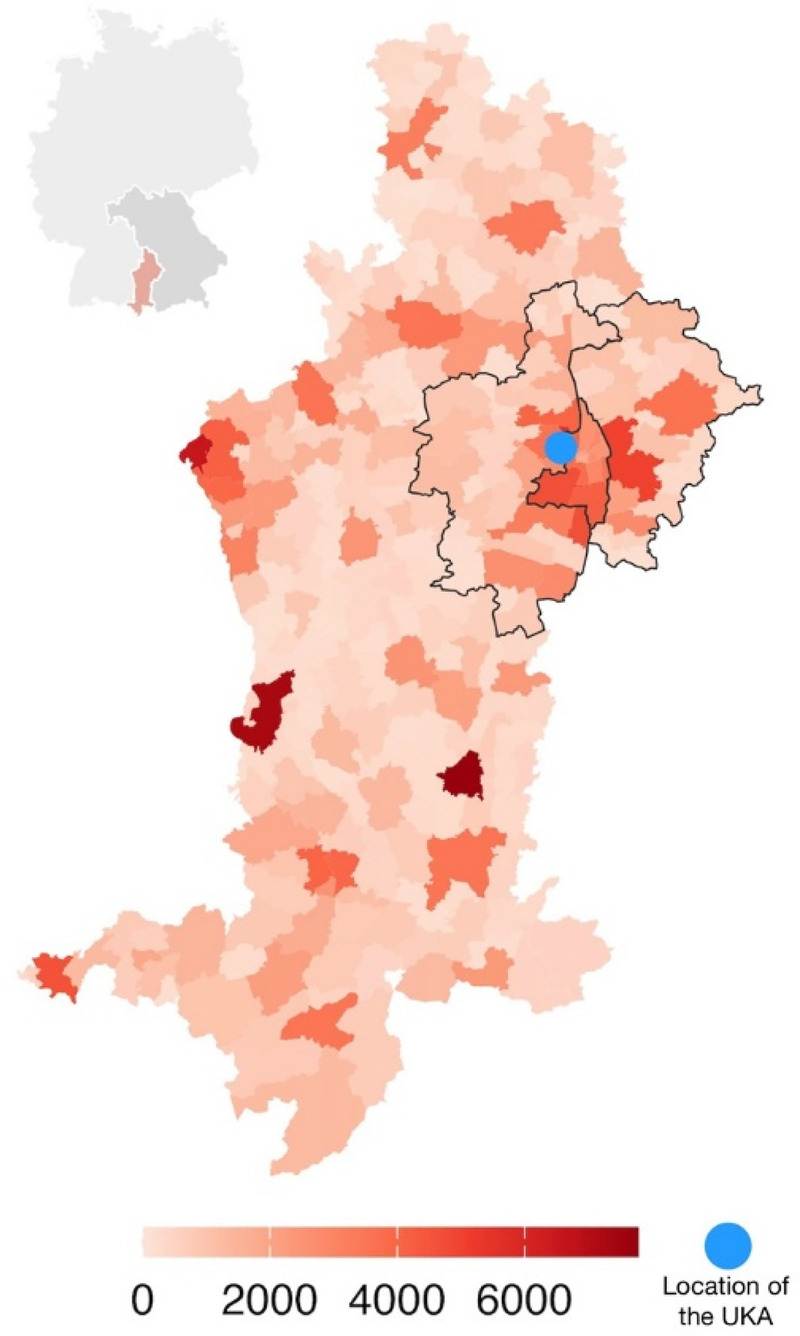
Geographic catchment area of the pediatric emergency department of university hospital Augsburg (UKA) and population density of children and adolescents aged 0–18 years in Bavarian Swabia. The map shows Augsburg and the surrounding counties in Bavarian Swabia, Germany, outlined in black, with the geographical distribution of the population aged 0–18 years per km². UKA is marked as a blue dot. Administrative boundary data were obtained from the Bavarian State Office for Survey and Geoinformation and adapted by the authors (ALKIS administrative boundaries; https://www.govdata.de/suche/daten/alkis-verwaltungsgrenzen251fa; CC BY 4.0: https://creativecommons.org/licenses/by/4.0/). Population data were obtained from the German Federal Statistical Office (Destatis), Census 2022, under the Data Licence Germany - Attribution - Version 2.0 (dl-de/by-2-0), and were processed and visualized by the authors.

The PED catchment area includes the city of Augsburg, with 301,000 residents as of December 2024, and the surrounding counties, extending across the administrative district of Bavarian Swabia, which had 1,933,313 residents as of December 2024 (Figure 1). Supplementary Figure 1 illustrates geographic access to G-BA-certified pediatric emergency care in Germany and Bavaria, including UKA. The figure shows travel time and distance to the nearest G-BA-certified pediatric emergency care unit, with magnified views of Bavaria and the Augsburg metropolitan area. Outpatient pediatric care alternatives within a radius of 25 km around Augsburg include more than 60 private pediatric practices and over five urgent care clinics operated by the Bavarian Association of Statutory Health Insurance Physicians (KVB) (26), the regional statutory physicians' association responsible for outpatient care provision in Bavaria.

### 2.2 Data source and study population

Electronic visit-level records were extracted from the hospital information system ORBIS (Dedalus Healthcare Group, Bonn, Germany) for all presentations to the PED between January 1, 2017, and December 31, 2024. Identifiable personal data were removed before analysis, and only pseudonymized records were used in accordance with applicable data protection regulations. All visits during the study period were included in the descriptive cohort analysis. The visit-level data set was ordered by date and time of presentation. Extracted variables included arrival date and time, weekday, weekend or holiday status, age, sex, chief complaint, time to triage, triage acuity, mode of transport, referral source, PED length of stay (LOS), discharge disposition, and left without being seen (LWBS) status.

Chief complaints were documented by nursing staff at presentation using the Canadian Emergency Department Information System classification (CEDIS) and grouped into 18 categories (27).

After registration, children and adolescents presenting to the PED were triaged by trained nursing staff according to local PED procedures. Time to triage was defined as the interval, in minutes, from arrival or registration to completion of triage. Triage acuity was assigned using the five-level Emergency Severity Index (ESI), an emergency department triage algorithm based on clinical acuity and anticipated resource use. ESI level 1 indicates the need for immediate life-saving intervention; level 2, a high-risk situation; level 3, an expected need for two or more resources; level 4, an expected need for one resource; and level 5, no expected resource use (28).

Mode of transport was categorized as self-transport or emergency-vehicle transport. Emergency-vehicle transport included ambulance, patient transport ambulance, emergency physician vehicle, intensive care transport vehicle, and rescue helicopter. Referral source was categorized as self-referral or referral by medical personnel, including hospital transfer, KVB urgent care clinic, hospital emergency practice, ambulance service, emergency physician, or primary-care pediatrician. PED LOS was defined as the interval, in minutes, from arrival to discharge or transfer. Disposition was categorized as outpatient or inpatient. Outpatient disposition included discharge home, referral to a primary-care physician, leaving against medical advice, or premature termination of the visit. LWBS was defined as leaving the PED after triage but before physician assessment.

### 2.3 Operational capacity and epidemiologic context

For the period from January 1, 2020, to December 31, 2024, shift-level nursing schedule data were available and linked to the visit-level data set at the day level. Staffing was assessed separately for early, late, and night shifts according to the local PED roster. The planned nursing staffing model was 4 nurses during the early shift (5:55 a.m.–2:05 p.m.), 4 during the late shift (1:25 p.m.–9:35 p.m.), and 3 during the night shift (9:00 p.m.–6:20 a.m.). During shift transitions, children and adolescents present in the PED were assigned according to the shift interval in which they were present, allowing overlap across adjacent shifts when a visit spanned more than one shift. For each shift, the difference between planned and observed nursing staff was calculated. A day was classified as understaffed if at least 1 nurse was missing from the planned schedule in any shift. A dynamic patient-to-staff ratio (ps-ratio) was calculated for each PED visit as the number of children and adolescents simultaneously present in the PED divided by the number of nurses on duty during the corresponding nursing shift. The resulting values were subsequently summarized at the shift and day levels. For each visit, presence in the PED was defined from the recorded time of arrival to the recorded time of discharge, transfer, or premature termination of the visit. Time-varying counts of children and adolescents present in the PED were reconstructed from these arrival and departure timestamps and linked to the nursing roster for the respective shift. The ps-ratio was therefore defined as: ps-ratio = number of concurrently present children and adolescents in the PED/number of nurses on duty. Shift-level ps-ratios were calculated separately for early, late, and night shifts; daily ps-ratio values were subsequently summarized as daily medians for the main day-level analyses. Staffing data reflected documented nominal nursing coverage per shift and did not capture staff breaks, short-term within-shift absences, individual professional experience, skill mix, or concurrent nonclinical duties. Accordingly, the ps-ratio was used as an operational measure of workload relative to available nursing staff.

Physician staffing data were not continuously available with the same temporal resolution as nursing coverage and could therefore not be analyzed as a separate dynamic operational variable. Although continuous pediatric physician coverage, including senior pediatric backup, was maintained throughout the observation period, the available data did not capture physician number, seniority, competing duties, consultation load, or specialty-specific availability at shift level. Specialty-specific surgical coverage was therefore considered part of the local staffing structure rather than a time-varying crowding variable.

Daily PED visit counts and absolute daily counts of positive respiratory virus tests performed in the PED were linked to the same day-level data set. Respiratory viruses included influenza A/B, SARS-CoV-2, human metapneumovirus, and respiratory syncytial virus. Viral activity was therefore operationalized as the number of documented positive tests per day among children and adolescents tested in the PED. Testing denominators, positivity rates, and standardized regional surveillance measures were not available in the routine dataset. Viral activity was included as an epidemiologic context variable because seasonal respiratory infections may affect PED volume, case mix, and resource use. Accordingly, respiratory virus data were interpreted descriptively as contextual indicators of infectious burden rather than as a standardized measure of community viral incidence.

The statutory COVID-19 lockdown period in Bavaria was not excluded from the primary analyses but was considered separately in descriptive and comparative analyses. This period was defined as March 21, 2020 (29), to March 28, 2021 (30) corresponding to the interval of strict legal restrictions in Bavaria. This approach was used because pandemic-related policy changes and altered care-seeking behavior may have affected PED census, staffing patterns, testing practices, and respiratory viral activity. Because the ps-ratio was available only from 2020 onward, analyses involving the ps-ratio included the lockdown period, with lockdown status examined descriptively to contextualize potential shifts PED visit volume and staffing patterns.

### 2.4 Data cleaning and preparation

**Figure 2:**
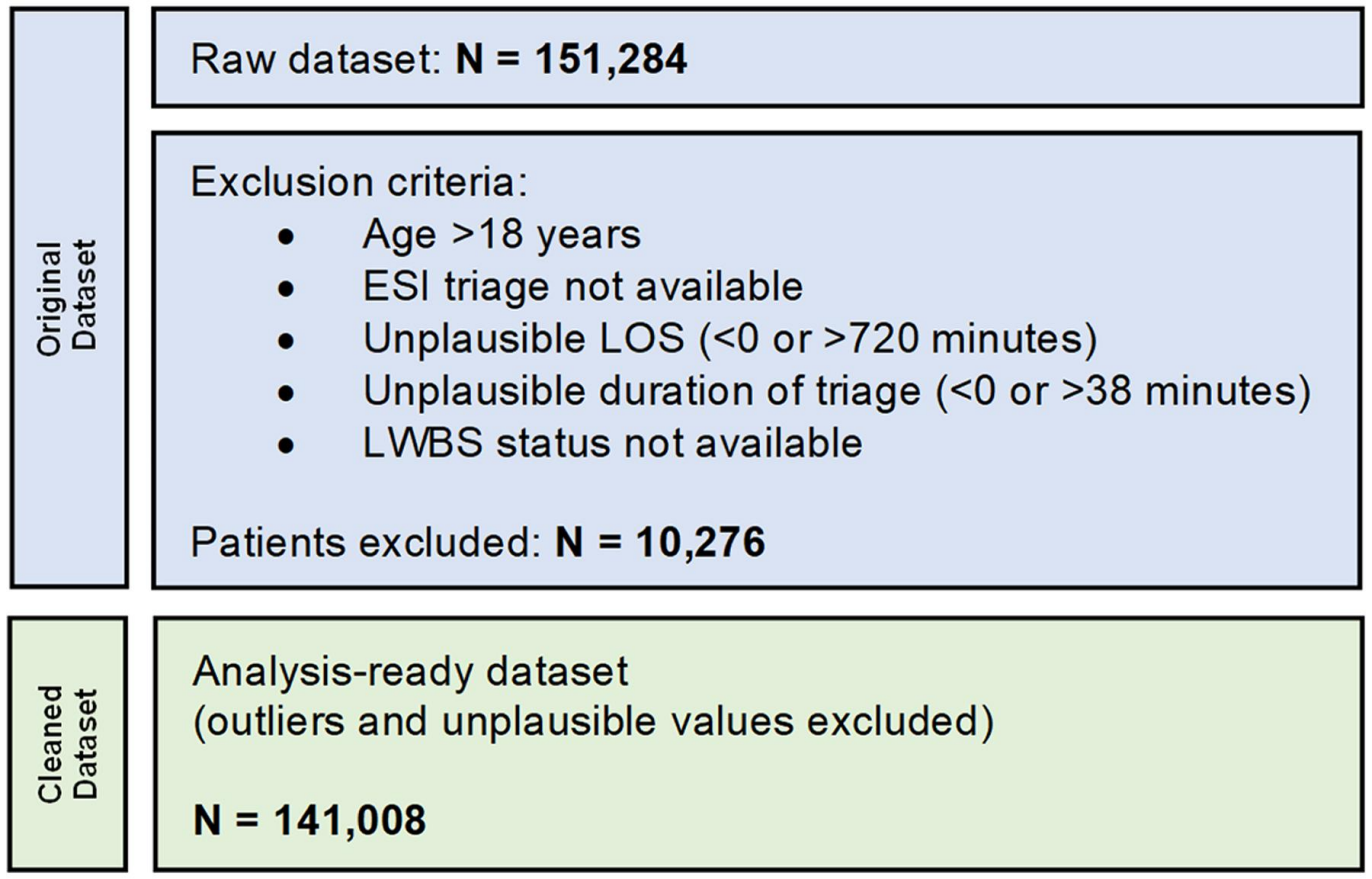
Selection of pediatric emergency department visit records for the final analytic cohort, 2017–2024. The flow diagram shows the number of extracted PED visit records and exclusions based on predefined criteria for implausibility, incompleteness, and non-recoverable missingness. The remaining records were included in the final analytic cohort and subsequent statistical analyses.

Data were screened for errors, implausible values, and missingness according to predefined rules. Exclusion criteria were predefined to identify records with non-recoverable missingness, structural implausibility, or likely administrative timestamp errors. The record-selection process is shown in Figure 2.

The thresholds for implausible LOS and time-to-triage values were applied to identify records likely affected by administrative or timestamp errors rather than to exclude clinically meaningful crowding episodes. In the local ORBIS workflow, very long apparent LOS values may occur when a visit is not administratively closed at the actual time of discharge or transfer, whereas implausible triage durations may reflect delayed documentation or incomplete timestamp capture. Because very long LOS and delayed triage may also reflect true operational strain, the effect of these data-cleaning exclusions was assessed in sensitivity analyses using the dataset before outlier exclusion but after removal of records with non-recoverable missingness or structural implausibility.

### 2.5 Cohort analysis and candidate overcrowding metrics

All visits between January 1, 2017, and December 31, 2024, were included in descriptive analyses. Candidate overcrowding metrics were identified through a literature-based review of measurement approaches used in adult EDs and PEDs, as previously detailed elsewhere (19). Four candidate parameters were evaluated as markers of overcrowding: (i) LWBS, defined as the absolute daily count (14, 31); (ii) PED LOS, measured in minutes (4, 17, 31, 32); (iii) time to triage, measured in minutes and interpreted with reference to the legal requirement that triage should be completed within 10 min (33); and (iv) the dynamic ps-ratio, available for 2020–2024.

Overcrowding parameters were primarily analyzed at the day level. This aggregation level was selected to harmonize all candidate metrics within a common temporal unit, to align LWBS events with daily operational and epidemiologic context variables, including absolute daily respiratory virus detections, and to reduce the influence of short-term documentation variability in routine timestamp data. LWBS was assessed as the absolute daily count, whereas the three continuous visit-specific parameters LOS, time to triage, and ps-ratio were summarized as daily medians. Statistical comparisons among candidate metrics were restricted to 2020–2024, the period in which all 4 parameters were available. The derivation of ps-ratio-based overcrowding thresholds used all available days from 2020 to 2024, including the COVID-19 lockdown period; lockdown status was examined descriptively to contextualize potential shifts in PED visit volume and staffing patterns.

Candidate metrics were assessed for plausibility, internal coherence, and correlation with the other parameters. Metrics were interpreted according to whether they primarily reflected concurrent workload and capacity or downstream consequences of crowding, such as delayed triage or prolonged LOS. Exploratory shift-level analyses were performed for selected operational parameters to provide a more granular description of nursing-shift-correlated crowding patterns and to assess whether the day-level findings were consistent with more episodic operational strain within nursing shifts. These included LWBS counts per nursing shift and absolute ps-ratio values by shift. Because not all candidate parameters and contextual variables were available or equally robust at the shift level, these analyses were considered supplementary and hypothesis-generating rather than the primary basis for threshold derivation.

### 2.6 Statistical analyses

As the data were not normally distributed, each continuous variable was presented as median along with the respective interquartile range in the form $\left(q_{25};q_{75}\right)$. Consequently, groups were compared using the non-parametric, two-sided Mann–Whitney U test. Categorical variables were given as absolute and relative frequencies and assessed applying the *χ*^2^-test. Both Pearson and Spearman's rank correlation coefficients were calculated to quantify the bivariate relationships between continuous variables.

Predicted probabilities derived from logistic regression models were used to construct raw receiver operating characteristic (ROC) curves for continuous overcrowding parameters. Daily LWBS events were operationalized as binary internal anchors using two thresholds: ≥1 and ≥2 LWBS events per day. To account for autocorrelation and temporal structure, adjusted ROC curves were constructed using segmented time-series regression as described below. The optimal local ps-ratio thresholds were identified using the Youden index (i.e., max(sensitivity+specificity−1)). Sensitivity analyses were performed using a dataset with outliers retained (see Section 2.4 and Figure 2).

As a part of a time series analysis, nonlinear temporal trends in overcrowding parameters were modeled using segmented generalized additive mixed models with penalized regression splines for calendar time and an AR(1) (autoregressive) correlation structure to account for autocorrelation. A lockdown indicator was included to estimate the level shift during the statutory COVID-19 lockdown period. Whilst autocorrelation was addressed in ROC analyses and longitudinal time-series models, the calculation of the correlation coefficients was considered as exploratory analysis at the day level. Statistical significance was defined based on a type I error of *α* = 0.05. All analyses were performed using the open-source statistical software R (version 4.5.1).

### 2.7 Ethics approval

This study was conducted in accordance with the ethical principles outlined in the Declaration of Helsinki (34). Ethical approval for this study was waived by the ethics committee of Ludwig-Maximilians-University Munich (LMU), Germany as it involved a retrospective analysis of routinely collected, pseudonymized data (ID 25-0057 K). Signed informed consent was not required, as the study used pseudonymized data and adhered to all applicable ethical standards.

## 3 Results

### 3.1 Descriptive characteristics of patient visits

From 151,284 extracted PED visits, 141,008 were included in the final analytic cohort after application of predefined exclusion criteria (Figure 2; Table 1), corresponding to a median of 18,337 visits per year during the study period (range, 13,623–20,393). Visit volume was disproportionately higher on weekends and holidays, accounting for 33.9% of total visits. Specifically, Saturdays and Sundays alone accounted for 15.4% and 15.2% of total visits, respectively. The median age of children and adolescents presenting to the PED was 5.5 years, and 55.9% of visits were by male children and adolescents. The leading CEDIS chief complaint categories were orthopedic or trauma-related presentations (23.3%), general or other complaints (18.2%), neurologic symptoms (12.9%), and gastrointestinal symptoms (12.5%). The most frequent individual chief complaints were fever, extremity injury, head injury, abdominal pain, and cough. Most visits were assigned ESI level 4 (58.1%). Presentation was predominantly self-directed: 86.5% of visits involved self-transport, and 78.3% occurred without external medical referral. Overall, 74.8% of visits resulted in outpatient disposition. The proportion of visits ending in LWBS was 2.1%.

**Table 1:** Baseline characteristics of pediatric emergency department visits included in the final analytic cohort at university hospital Augsburg, 2017–2024.

Characteristic	Total (*n*)	*n* (%) or median (IQR)
Weekday	1,41,008	
Monday		19,944 (14.1%)
Tuesday		19,105 (13.5%)
Wednesday		19,296 (13.7%)
Thursday		19,107 (13.6%)
Friday		20,411 (14.5%)
Saturday		21,779 (15.4%)
Sunday		21,366 (15.2%)
Weekend or holiday	1,41,008	
False		93,168 (66.1%)
True		47,840 (33.9%)
Age (years)	1,41,008	5.55 (2.158; 11.261)
Sex	1,40,993	
Female		62,214 (44.1%)
Male		78,779 (55.9%)
CEDIS chief complaint	1,41,008	
General and minor		25,686 (18.2%)
Ophthalmology		6,500 (4.6%)
Gastrointestinal		17,621 (12.5%)
OB/GYN		285 (0.2%)
Skin		6,934 (4.9%)
ENT—mouth, throat, neck		3,845 (2.7%)
ENT—nose		1,466 (1%)
ENT—ears		2,488 (1.8%)
Cardiovascular		1,883 (1.3%)
Neurologic		18,209 (12.9%)
Orthopedic		32,912 (23.3%)
Mental health		131 (0.1%)
Respiratory		10,153 (7.2%)
Substance misuse		1,130 (0.8%)
Other trauma		219 (0.2%)
Environmental		356 (0.3%)
Unknown		7,312 (5.2%)
Genitourinary		3,878 (2.8%)
Leading compliants	1,41,008	
Fever		13,180 (9.3%)
Extremity injuries		11,740 (8.3%)
Head injury		7,922 (5.6%)
Abdominal pain		10,135 (7.2%)
Cough		5,681 (4%)
Time to triage (minutes)	1,41,008	10 (5; 16)
ESI triage level	1,41,008	
1		587 (0.4%)
2		14,012 (9.9%)
3		30,469 (21.6%)
4		81,946 (58.1%)
5		13,994 (9.9%)
Mode of transport	1,41,008	
Self transport		122,009 (86.5%)
Emergency transport		18,999 (13.4%)
Referral type	1,41,008	
Self referral		110,387 (78.3%)
Physician/Paramedic referral		30,621 (21.7%)
Length of stay (minutes)	1,41,008	114 (68; 175)
Discharge dispotion	1,41,008	
Outpatient		105,498 (74.8%)
Inpatient		35,509 (25.2%)
Information on discharge not available		1 (0%)
Left without being seen	1,41,008	
False		138,103 (97.9%)
True		2,905 (2.1%)

Data are presented as absolute (relative) frequencies for categorical variables and median (IQR) for continuous variables.

### 3.2 Operational capacity and epidemiologic context

**Figure 3:**
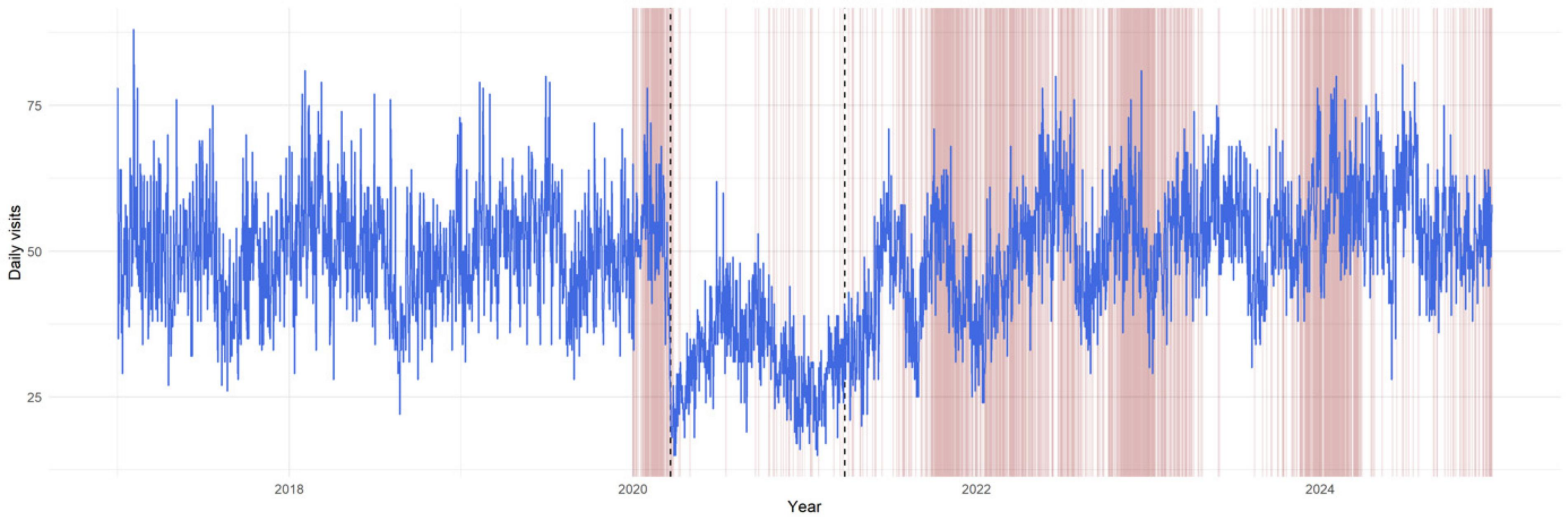
Daily pediatric emergency department visit volume and respiratory viral activity at university hospital Augsburg, 2017–2024. Daily PED visit counts are shown across the full observation period. The statutory COVID-19 lockdown period in Bavaria is indicated by vertical dashed lines. Days with at least 1 positive respiratory virus test documented in the PED are color-coded by vertical lines in matte dark red.

From 2020 to 2024, the median number of daily PED visits was 51 (Table 2). During the statutory COVID-19 lockdown period, the median daily census decreased to 31 visits. Nursing understaffing was common: at least 1 shift was understaffed on 91.6% of days and on 98.9% of days during the lockdown period. Across all nursing shifts, 38.0% were understaffed, with shortages occurring most often during early and late shifts and least often during night shifts (Supplementary Figure S2). Daily PED visit volume, nursing staffing patterns, and respiratory viral activity varied across the observation period (Figure 3). Respiratory virus detections showed distinct temporal clustering, occurring alongside fluctuations in PED visit volume.

**Table 2:** Daily nursing operational capacity, respiratory viral activity, and candidate overcrowding metrics in the pediatric emergency department of university hospital Augsburg, 2020–2024, stratified by statutory COVID-19 lockdown period.

Characteristics	Non-lockdown period	COVID-19 lockdown period	*p*-value
Staff			
No understaffing	122 (8.4%)	4 (1.1%)	<0.001
Understaffing	1,333 (91.6%)	368 (98.9%)	<0.001
Daily PED visits	51 (44; 57)	31 (26.75; 37)	<0.001
LWBS	1 (0; 2)	0 (0; 1)	<0.001
Time to triage (minutes)	10 (8; 11)	10 (9; 11.5)	0.008
LOS (minutes)	114 (98; 131.5)	108 (93; 125.625)	<0.001
ps-ratio	2.333 (2; 3)	1.667 (1.333; 2)	<0.001

Data are presented as absolute (relative) frequencies for categorical variables and median (IQR) for continuous variables. *P*-values compare the pre-/post-lockdown period with the lockdown period. Nursing understaffing was defined as at least 1 nurse missing from the planned nursing shift schedule.

### 3.3 Overcrowding parameters

Across the entire observation period, 2.1% of all PED visits resulted in LWBS (Table 1). From 2020 to 2024, the median LWBS count was 1 visit per day; during the COVID-19 lockdown period, this decreased to 0 visits per day (Table 2). In supplementary exploratory shift-level analyses, the mean LWBS count was 0.33 visits per nursing shift, with temporal variation across shifts and years (Supplementary Figure S3).

**Figure 4:**
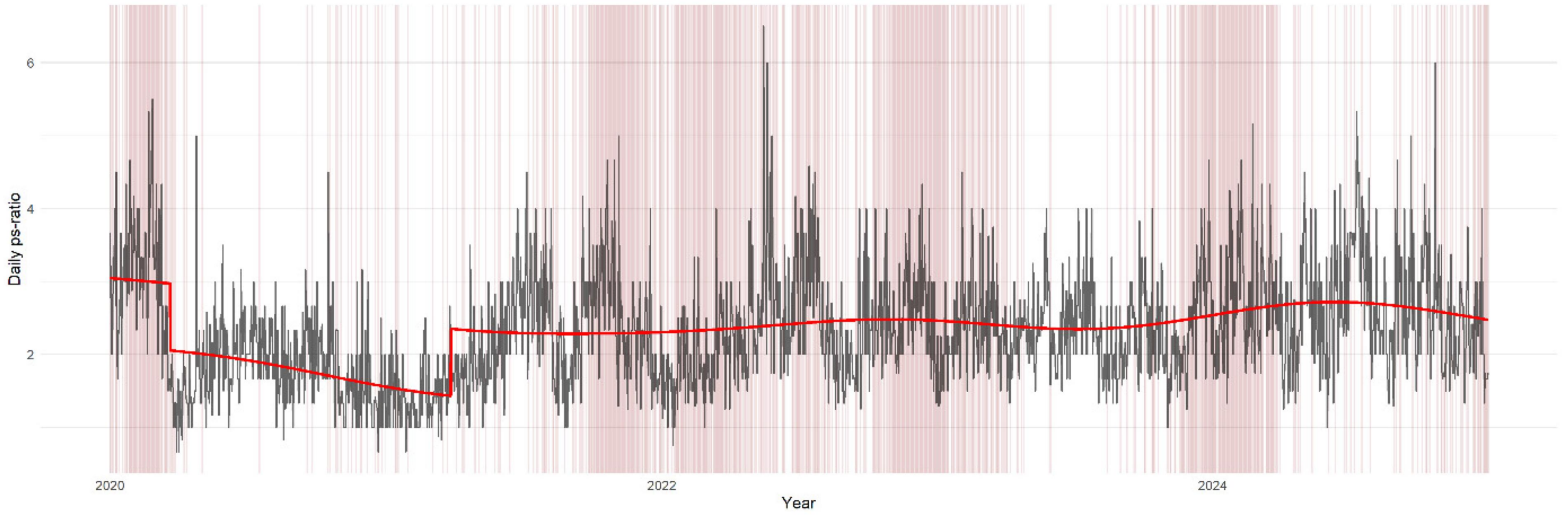
Daily patient-to-staff ratio in the pediatric emergency department of university hospital Augsburg, 2020–2024. The figure shows the time course of the median daily patient-to-staff ratio (ps-ratio), defined as the number of children and adolescents simultaneously present in the PED divided by the number of nurses on duty. The ps-ratio is shown as a staffing-sensitive measure of operational strain over time. Days with at least 1 positive respiratory virus test documented in the PED are color-coded by vertical lines in matte dark red. The red line shows the non-linear temporal trend of the ps-ratio.

The median LOS was 114 min across the full cohort and 108 min during the COVID-19 lockdown period. Time to triage showed little variation at the aggregate level, with a median of 10 min both across the full observation period and during the lockdown interval. The ps-ratio was lower during the COVID-19 lockdown period than during the pre- and post-lockdown periods, with a median of 1.66 compared with 2.33 children and adolescents per nurse (Table 2). This decrease paralleled the reduction in daily PED presentations during the lockdown period (Figure 3). Time-series analysis demonstrated a nonlinear ps-ratio trend over time (effective degrees of freedom, 6.3; *P* < .001), with a level shift of −0.92 during the COVID-19 lockdown period and moderate autocorrelation (*ρ* = 0.32) (Figure 4).

**Figure 5:**
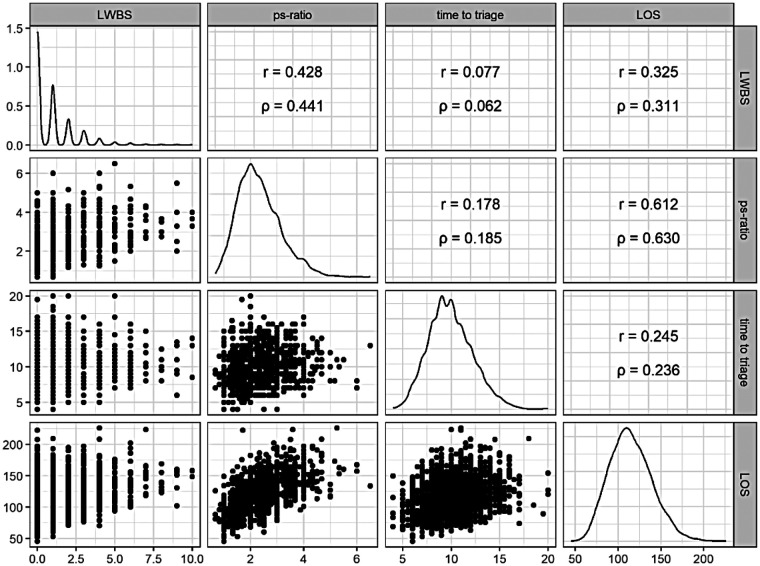
Correlation matrix of day-level candidate overcrowding parameters in the pediatric emergency department of university hospital Augsburg, 2020–2024. The figure shows pairwise day-level relationships (*n* = 1,827 days) among the four candidate overcrowding parameters available during the period with nursing-staffing data. Parameters included the daily number of visits ending in left without being seen (LWBS), the daily median patient-to-staff ratio (ps-ratio; number of children and adolescents simultaneously present in the PED divided by the number of nurses on duty), daily median time to triage in minutes, and daily median length of stay (LOS) in minutes. Kernel-density distributions of each parameter are shown on the diagonal, bivariate scatterplots are shown in the lower panels, and Pearson correlation coefficients (*r*) and Spearman rank correlation coefficients (*ρ*) are shown in the upper panels.

Correlation analyses were restricted to 2020–2024, when all 4 candidate overcrowding parameters were available. All candidate parameters showed positive correlations with each other (Figure 5). Correlations were based on daily medians for LOS, time to triage, and ps-ratio, and on absolute daily counts for LWBS. The strongest correlation was observed between ps-ratio and LOS (Pearson's *r* = 0.612, Spearman's *ρ* = 0.630). LWBS was also positively correlated with ps-ratio (*r* = 0.428, *ρ* = 0.441). Time to triage showed the weakest correlations with the other parameters.

ROC analyses using LWBS of at least 1 or 2 visits per day as the binary reference identified ps-ratio cutoffs between 2.03 and 2.20 as the optimal thresholds for overcrowding (Table 3). In the adjusted ROC analysis based on the multivariable time-series model (Table 3, accounted for autocorrelation, non-linear trend, and the COVID-19 period), the LWBS ≥2 anchor yielded the highest AUC of 0.76, with sensitivity and specificity both equal to 0.70. Across the period with available ps-ratio data from 2020 to 2024, 1,827 days were analyzed for exceedance of the locally derived ps-ratio threshold. During this period, positive detections of respiratory viruses with available or emerging vaccination or immunoprophylaxis strategies were frequently observed and showed temporal overlap with periods of ps-ratio threshold exceedance. Specifically, RSV was detected on 790 occasions, influenza on 384 occasions, and SARS-CoV-2 on 655 occasions. Because several positive tests and more than one virus detection could occur on the same day, these virus-specific counts were not mutually exclusive and were interpreted as descriptive indicators of infectious burden rather than as distinct crowding episodes or evidence of a causal association between viral activity and overcrowding.

**Table 3:** Receiver operating characteristic (ROC) analyses of the patient-to-staff ratio as a candidate overcrowding marker using daily left-without-being-seen events as internal reference anchors, 2020–2024.

	ROC analysis (raw)		ROC analysis (adjusted)	
Reference	LWBS ≥ 1	LWBS ≥ 2	LWBS ≥ 1	LWBS ≥ 2
AUC	0.73	0.73	0.75	0.76
Optimal cut-off (Youden index)	2.15	2.21	2.03	2.20
Sensitivity	0.69	0.76	0.72	0.70
Specificity	0.66	0.58	0.67	0.70

Raw and adjusted ROC curves were derived from univariable logistic regression and multivariable segmented autoregressive AR(1) time-series regression, respectively. The AR(1) models were adjusted for non-linear temporal trends and segmented into lockdown and non-lockdown periods. Daily left-without-being-seen (LWBS) events, defined as either ≥1 or ≥2 LWBS events per day, were used as binary internal reference anchors.

### 3.4 Sensitivity and supplementary shift-level analyses

Distribution of ESI categories across nursing shifts is presented in Supplementary Table S1; no statistically significant differences were observed. Further exploratory shift-level operational analyses are shown in Supplementary Figures S3–S6. Absolute LWBS counts by shift, median ps-ratio by shift, and visit counts by shift over time are provided in Supplementary Figures S3–S5, respectively, and pairwise correlations among shift-level overcrowding parameters are shown in Supplementary Figure S6. Overall, these supplementary shift-level analyses were consistent with the day-level findings, demonstrating similar positive relationships among ps-ratio, LWBS events, and process times. In sensitivity analyses, the dataset before outlier exclusion but after removal of records with non-recoverable missingness or structural implausibility comprised 150,956 visits. Results based on this dataset were consistent with the main analyses (Supplementary Figure S7, Supplementary Tables S2, S3).

## 4 Discussion

This study aimed not only to compare candidate indicators of pediatric emergency department (PED) overcrowding, but to derive a local operational definition of overcrowding that emerges from the specific care environment of our PED. Three main messages arise from our findings. First, our data suggest that overcrowding in pediatric emergency care is influenced by local structures of care and the way demand presents in each setting. Second, pediatric overcrowding is temporally dynamic and shaped by external pressures such as infectious waves and pandemic-related shifts in care-seeking behavior. Third, among the evaluated parameters, the patient-to-staff ratio (ps-ratio) emerged as the most actionable marker, as it most directly reflects the real-time relationship between patient load and available nursing capacity.

### 4.1 PED overcrowding is shaped by local demand patterns and care structures

The descriptive cohort characteristics indicate a nonuniform temporal demand pattern, with a substantial proportion of PED visits occurring during weekends and holidays. This pattern may reflect limited availability of outpatient pediatric services outside regular office hours and supports the role of the PED as both an emergency care provider and an access point for unscheduled pediatric care. Similar weekend effects have been reported in some pediatric ED cohorts (35), whereas other studies observed higher attendance during weekdays (36), underscoring that pediatric emergency demand is context-sensitive and shaped by local care structures and access pathways.

In Augsburg, although statutory outpatient pediatric services and KVB urgent care structures are available, differences in opening hours, perceived accessibility, and parental care-seeking preferences may shift low-acuity demand toward the PED, particularly outside regular office hours. Additionally, increased leisure activities on weekends could contribute to injury-related visits. In this sense, the weekend effect in our cohort may be part of the local background against which overcrowding must be interpreted.

Male patients were overrepresented, consistent with other pediatric ED cohorts (35, 36). This pattern may reflect sex-related differences in injury risk or care-seeking behavior, but it is unlikely to be a primary driver of the operational overcrowding phenotype examined here. Adult ED studies may show different sex distributions and temporal trends, but these should be interpreted cautiously in the pediatric setting (37).

The distribution of CEDIS chief complaints, including the 5 most frequent categories, was broadly consistent with data from the 2024 German AKTIN Emergency Department Registry (38), supporting the plausibility of the OACS+ cohort while underscoring the need to interpret local crowding within local care structures.

The predominance of ESI level 4 presentations, together with the high proportion of self-referred and self-transported patients, indicates that much of the observed PED demand arose from lower-acuity, unscheduled presentations. Although such cases may require limited resources individually, their cumulative burden can be substantial because they occupy waiting capacity, treatment space, nursing attention, and physician time. Thus, pediatric overcrowding in this setting was not driven solely by critically ill patients but also by the aggregate operational load generated by many lower-acuity visits (31, 39). This interpretation is supported by Sorz-Nechay et al. (40), who described the tertiary PED at the Department of Pediatrics and Adolescent Medicine at the Medical University Vienna, Austria in which 95% of visits were self-referred and approximately 75% were classified as low urgency according to the Manchester Triage System (MTS; urgency levels 4 and 5) (41), with this patient mix contributing to triage delays, overcrowding in waiting areas, limited room availability, and staff exhaustion. Importantly, the same study showed that a structured MTS-based referral pathway to pediatric offices and secondary-care hospitals was correlated with a marked reduction in total visits, suggesting that the burden created by low-acuity presentations is not only descriptive but potentially modifiable when local ambulatory alternatives and referral pathways are available (40). The study further underscores that redirection strategies require structured workflows, defined exclusion criteria, and clear communication with families, indicating that local crowding interventions are organizational and communicative as well as logistical (40).

In our cohort, most PED visits resulted in outpatient management, including discharge home, referral to a primary-care physician, or leaving against medical advice, while 25.2% resulted in inpatient admission after PED evaluation. Admission rates in pediatric ED cohorts are often lower than in adult EDs, reflecting a higher proportion of non-urgent presentations (42, 43). Published pediatric ED admission rates frequently range between 10% and 20% (42–44), which is lower than observed in our cohort. However, the 2024 German AKTIN Emergency Department Registry reported an inpatient admission proportion of approximately 21%, which is broadly comparable to the admission rate observed in our cohort (38).

Taken together with the predominance of lower-acuity triage categories and the high proportion of ambulatory disposition, these findings indicate that our PED serves not only as a site for acute tertiary care, but also as an important access point for unscheduled pediatric care. This local role is essential for interpreting overcrowding in our setting.

Analysis of the 2020–2024 staffing schedules showed that planned nursing coverage of the ED was unmet most of the time, with even more understaffing during the COVID-19 period. This underlines the widespread nursing shortage in Germany (45), which is also observed internationally (46) and its potential negative impact on care quality and overcrowding. These findings emphasize that overcrowding cannot be inferred from patient volume alone, but arises from the interaction between demand, staffing, and available space.

### 4.2 Pediatric overcrowding is temporally dynamic and influenced by infectious and pandemic-related demand shifts

Daily PED visit counts fluctuated around 50 and showed a modest rising trend, interrupted by a decline during the COVID-19 lockdown period. This decline may reflect lockdown measures, infection concerns, and altered care-seeking behavior, as reported in pediatric ED studies during the COVID-19 pandemic. The concurrent reduction in LOS may reflect lower patient volume, altered case mix, changes in care-seeking behavior, or pandemic-related workflow adaptations, rather than a single mechanism (47). These findings indicate that pediatric overcrowding in this setting fluctuated over time and coincided with broader epidemiologic and societal conditions. This is particularly relevant in pediatric emergency care, where respiratory infectious waves may alter attendance patterns, case mix, and operational strain (48, 49). Viral activity and pandemic-related disruptions should therefore be considered part of the local context in which overcrowding emerges.

The decline in PED visits and in the ps-ratio during the COVID-19 period, despite persistent understaffing, suggests that crowding levels may change substantially when demand patterns shift. Consistent with findings in adult emergency departments, these observations support the interpretation that overcrowding reflects an interaction between available resources and temporally fluctuating demand, rather than a fixed departmental property (50). The temporal dimension is therefore central to deriving a local operational definition of overcrowding, and any marker intended to support predictive modeling and intervention studies must remain meaningful during periods of fluctuating disease incidence and changes in care-seeking behavior. This was also reflected in the observed temporal clustering of ps-ratio increases with periods of RSV, influenza, and SARS-CoV-2 activity, indicating that respiratory infectious burden formed part of the local operational context of PED crowding. Because prevention strategies for these infections differ by age group, risk profile, season, and calendar period, future studies linking individual-level immunization status, disease severity, testing activity, and operational strain may help determine whether targeted respiratory infection prevention strategies can strengthen PED resilience (51).

### 4.3 The ps-ratio is the most operationally meaningful marker because it reflects real-time strain rather than only downstream consequences

The correlation analyses showed that time to triage had the weakest correlation with the other parameters, suggesting that it was not a sensitive stand-alone marker of overcrowding in this cohort. The median time to triage duration remained consistently at 10 min throughout the observation period, thus meeting the statutory requirements of the German Federal Joint Committee (G-BA).

Previous pediatric literature has considered triage waiting time as one relevant operational dimension when describing crowding, although not necessarily as a stand-alone marker. In a tertiary Canadian PED, Stang et al. identified “wait at triage” as a distinct component of the overcrowding construct and concluded more broadly that operational-process and patient-volume measures were more relevant in pediatrics than markers of inpatient transfer delay (52). A broader systematic review of ED crowding literature likewise reported triage delays during crowded periods, although this review was not restricted to pediatric-only settings (12). Against this background, our results suggest that in this PED, time to triage was only minimally correlated with LOS, LWBS, and the patient-to-staff ratio.

These findings suggest that local triage processes remained comparatively stable even under varying levels of operational strain; time to triage therefore appeared to function more as a protected process metric than as a sensitive indicator of overall overcrowding.

The ps-ratio showed the strongest associations with the other parameters, most notably LOS and LWBS. These findings support the ps-ratio as the preferred operational marker in this cohort, not merely because of its correlation structure, but because it directly links patient demand to immediately available nursing capacity. By contrast, LOS and LWBS represent downstream consequences of crowding. Although both are widely used to describe overcrowding in adult and pediatric EDs (4, 14, 17, 31, 32), the relevance of the ps-ratio in our cohort is not only statistical. Pediatric emergency care literature increasingly suggests that staffing-sensitive measures are closely linked to operational performance. In an administrative data study from a PED, Janhunen et al. reported that nurse-patient ratios affected care processes and that higher nurse staffing levels were negatively related to emergency department length of stay (53). Similarly, Keijzers et al. showed that introducing a dedicated pediatric team during periods of peak workload in a mixed emergency department reduced total length of stay for pediatric patients (54), and Li et al. found a decrease in the length of stay with the implementation of a pediatric triage team in selected acuity groups (55). Together, these studies support the broader concept that staffing levels and staff organization meaningfully influence PED throughput. Against this background, our finding that the ps-ratio was more closely correlated with LOS and LWBS than the other examined parameters suggests that, in this PED, the balance between patient load and immediately available nursing capacity may capture operational strain more effectively than downstream outcomes alone. At the same time, this should be interpreted as a local operational finding rather than validation of a universally applicable staffing threshold, because pediatric crowding metrics remain heterogeneous across settings.

To our knowledge, few pediatric overcrowding studies have evaluated a dynamic patient-to-staff ratio as a locally derived operational marker.

In the present cohort, ROC analyses identified ps-ratio thresholds between 2.03 and 2.21 patients per nurse, depending on the LWBS anchor and model specification. Discrimination was moderate and remained broadly stable when LWBS was operationalized as either ≥1 or ≥2 events per day. For operational interpretation, a rounded threshold of approximately 2.2 patients per nurse provides a pragmatic local benchmark. This threshold should be interpreted as locally derived and exploratory rather than as an established definition of PED overcrowding. The threshold may support translation of a continuous staffing-sensitive measure into an operational decision point for the present care environment, but prospective validation is required before use as a clinical escalation trigger (56). The ps-ratio should also be interpreted in the context of its construct limitations. Although it captures a personnel-sensitive dimension of crowding by relating patient census to available nursing capacity, it should be interpreted as a staffing-sensitive operational marker rather than a complete measure of ED crowding. In particular, output constraints such as access block and boarding may independently drive overcrowding even when staffing ratios appear acceptable (57). The ps-ratio also does not directly account for other structural and flow-related constraints, such as treatment-room occupancy, physician workload, inpatient bed availability, diagnostic delays, consultation delays, or family-related spatial constraints. These unmeasured factors may contribute to the moderate association between ps-ratio and LWBS and underscore that this single staffing-sensitive metric cannot fully capture the multidimensional construct of PED overcrowding.

From an implementation perspective, locally derived crowding thresholds may help translate retrospective operational analyses into prospective capacity-management strategies. Once externally and prospectively validated, such thresholds could inform predefined escalation triggers for staffing reinforcement, activation of fast-track processes, patient redirection to appropriate outpatient structures, or structural flow interventions aimed at reducing access block (5, 7, 40, 56). However, these applications were not tested in the present study and should be evaluated prospectively before implementation in routine care.

## 5 Strengths and limitations

This study has several strengths. First, it is based on a large, real-world PED cohort covering 141,008 visits over an 8-year period, providing a robust empirical basis for characterizing local demand patterns and candidate overcrowding parameters. Second, the analysis combined visit-level clinical and operational data with high temporal resolution, including arrival and discharge times, triage timing, LOS, LWBS events, nursing shift schedules, and day-level counts of positive respiratory virus tests. This allowed overcrowding to be examined not only as a static aggregate outcome, but as a dynamic interaction between patient demand and available staffing resources, interpreted in the context of respiratory viral activity. Third, the study evaluated several candidate overcrowding metrics within the same care environment, enabling direct comparison of their internal coherence and operational plausibility. Fourth, the inclusion of shift-level supplementary analyses provided additional granularity and supported the day-level findings, although these analyses were not the primary focus of the present study.

However, several limitations should be considered. First, no universally accepted gold standard exists for defining overcrowding in PEDs. Accordingly, the use of LWBS in the ROC analyses should be regarded as an internal operational anchor rather than a standardized reference definition of overcrowding. LWBS is clinically relevant but may also be influenced by acuity distribution, parental care-seeking behavior, waiting-room conditions, communication, and local administrative processes. Therefore, the derived ps-ratio threshold and corresponding sensitivity and specificity estimates should be interpreted as exploratory and context-specific.

Second, the analyses were retrospective and based on data from a single tertiary PED. The proposed ps-ratio threshold was therefore derived within one specific local care environment. This limitation is consistent with the conceptual aim of the study, which was to derive a local operational definition of overcrowding, but external validation will be required before transferability to other PEDs can be assessed. Although the ps-ratio captured the balance between patient load and nursing capacity in the present PED, it should be interpreted as a staffing-sensitive operational marker rather than a complete measure of ED crowding, because output constraints such as access block and boarding may independently drive overcrowding even when staffing ratios appear acceptable (57).

Third, part of the extracted dataset had to be excluded because of implausible values or missing information. Several variables were documented during routine clinical care, which may have introduced documentation errors, timestamp inaccuracies, or delays, particularly during periods of high workload. Although exclusion thresholds were applied to reduce the effect of likely administrative timestamp errors, very long LOS or delayed triage can also represent true operational strain. Excluding such values may therefore have attenuated the most severe crowding episodes and biased estimates toward less extreme operational conditions.

Fourth, the primary analyses were conducted at the day level. Daily aggregation allowed harmonization of candidate metrics with operational and epidemiologic context variables, but may have obscured shorter peak crowding periods occurring within specific shifts or hours. Supplementary shift-level analyses provided additional granularity and were broadly consistent with the day-level findings, but they remained exploratory and did not replace a fully time-resolved crowding analysis.

Fifth, not all structural and process-related dimensions of overcrowding could be incorporated. The ps-ratio does not directly account for treatment-room occupancy, physician workload, diagnostic or consultation delays, inpatient bed availability, boarding, or family-related spatial constraints. Staffing-based analyses were available only from 2020 to 2024 and were based on nominal nursing shift coverage. They did not capture staff breaks, sick leave, temporary staff, skill mix, professional experience, team composition, or concurrent nonclinical duties. Physician staffing data were not continuously available at comparable temporal resolution and could therefore not be analyzed as a separate dynamic operational variable. Moreover, objective operational strain and staff-perceived workload may not fully overlap. A recent PED study found only weak correlations between the National Emergency Department Overcrowding Score (NEDOCS) and individual staff-reported workload, with better agreement during periods of higher crowding (58). Future studies should examine whether locally derived measures such as the ps-ratio align with perceived burden among nurses and physicians and whether they can support ethically acceptable forecasting, escalation, triage, or diversion strategies in pediatric emergency care.

Finally, respiratory viral burden was incorporated as a contextual variable only. Virus data were based on absolute positive test counts from routine PED testing. Testing denominators, positivity rates, and standardized regional surveillance measures were not available, and testing practices likely changed during and after the COVID-19 pandemic. In addition, the present analysis did not assess individual vaccination or immunoprophylaxis status, age-specific eligibility, risk-group indications, or changes in prevention recommendations over time. This is particularly relevant for RSV, influenza, and SARS-CoV-2, for which prevention strategies differ by age group, risk profile, season, and calendar period. Therefore, the observed overlap between respiratory virus detections and ps-ratio threshold exceedance should be interpreted as a hypothesis-generating ecological observation rather than evidence that immunization or immunoprophylaxis strategies directly modified PED overcrowding in this cohort (57).

## 6 Conclusion

Pediatric overcrowding is best interpreted in relation to the local structures of care and temporal demand patterns in which it occurs. In this cohort, the ps-ratio most directly captured the interaction between patient load and available nursing capacity and showed the strongest bivariate correlations with other indicators of operational strain. This work does not propose a universal threshold, but demonstrates the feasibility of moving from descriptive crowding indicators toward a context-grounded operational benchmark for a specific PED setting. The proposed ps-ratio threshold should therefore be interpreted as a locally derived exploratory benchmark that requires external validation, prospective testing, and evaluation of clinical applicability before implementation in other settings.

## Data Availability

The original contributions presented in the study are included in the article/Supplementary Material, further inquiries can be directed to the corresponding author.
