# Supplementary material for: A local operational definition of pediatric emergency department overcrowding: an 8-year retrospective observational cohort study from the OACS+ project
Source: Front Pediatr. 2026 Aug 5;14:1882596. doi: 10.3389/fped.2026.1882596 (PMC13485882; doi:10.3389/fped.2026.1882596)
Supplement: Supplementary file 1 [file Datasheet1.docx]

**Supplementary Materials**

**A local operational definition of pediatric emergency department overcrowding: an 8-year retrospective observational cohort study from the OACS+ project**

Johanna T. Meßner^1^, Dennis Freuer^2^, Christine Meisinger^2^, Birgit Prodinger^3^, Bin Zhou^4^, Melanie L. Conrad^5^, Anne Ch. Garbe^5^, Anna-Lena Behr^5^, Anna Juraschek^1^, Fabian B. Fahlbusch^5,6^, Florian Weber^1*^

^1^Interdisciplinary Pediatric Emergency Department, Faculty of Medicine, University of Augsburg, Augsburg, Germany; ^2^ Epidemiology, Faculty of Medicine, University of Augsburg, University Hospital Augsburg, Augsburg, Germany; ^3^Chair of Inclusive Health Care, Faculty of Medicine, University of Augsburg, Augsburg, Germany; ^4^Chair of Model-based Environmental Exposure Science, Environmental Health Sciences Institute, Faculty of Medicine, University of Augsburg, Augsburg, Germany; ^5^Neonatology and Pediatric Intensive Care, Faculty of Medicine, University of Augsburg, Augsburg, Germany; ^6^Bayerisches Zentrum für präventive Infektionsmedizin (BZI), Department of Pediatrics and Adolescent Medicine, Medical Faculty, University of Augsburg, Augsburg, Germany.

**Figure S1. Geographic Access to G-BA–Certified Pediatric Emergency Care in Germany and Bavaria.** Heatmap showing travel time to the nearest pediatric emergency care unit certified by the Federal Joint Committee (Gemeinsamer Bundesausschuss, G-BA) in Germany, with enlarged views of Bavaria and the Augsburg metropolitan area. The dashed circle centered on Augsburg has a diameter of 30 km and illustrates the transition from the metropolitan to the surrounding rural catchment area. The Bavarian panel shows the proportion of the population aged younger than 18 years by distance category to the nearest certified pediatric emergency care unit. Modified by the authors from the Krankenhausatlas of the Statistical Offices of the Federation and the Länder, Germany, licensed under the Data Licence Germany – Attribution – Version 2.0 (dl-de/by-2-0; https://www.govdata.de/dl-de/by-2-0); source: https://krankenhausatlas.statistikportal.de/; accessed April 23, 2026. Background map: basemap.de. Display settings: Bavaria; G-BA-certified pediatric emergency care. Data refer to hospitals participating in inpatient care as of December 31, 2023, including hospitals certified under the G-BA module for pediatric emergency care pursuant to §136c(4) of the German Social Code, Book V (SGB V).

**Figure S2. Nursing staffing deficits by shift in the pediatric emergency department, 2020-2024.** The figure shows the distribution of differences between observed and planned nursing staff per shift, stratified by early, late, and night shifts. The x-axis indicates the actual-minus-planned staffing difference for each shift; 0 denotes that planned staffing was achieved, whereas negative values indicate understaffing, with -1 to -4 corresponding to 1 to 4 fewer nurses than planned. The y-axis shows the number of nursing shifts in each staffing category.

**Figure S3. Shift-level counts of visits ending in left without being seen in the pediatric emergency department, 2020-2024.** The figure shows the number of visits ending in left without being seen (LWBS) per nursing shift over calendar time. Blue vertical bars indicate the absolute LWBS count for each nursing shift. Days with at least 1 positive respiratory virus test documented in the PED are shown as vertical lines in matte dark red. The vertical dashed lines indicate the statutory COVID-19 lockdown period in Bavaria. The y-axis shows the number of LWBS events per nursing shift.

**Figure S4. Shift-level patient-to-staff ratio in the pediatric emergency department, 2020-2024.** The figure shows the median patient-to-staff ratio (ps-ratio) per nursing shift over calendar time. The ps-ratio was defined as the number of children and adolescents simultaneously present in the PED divided by the number of nurses on duty during the corresponding shift. Grey vertical lines indicate shift-level ps-ratio values, and the red line shows the nonlinear temporal trend. Days with at least 1 positive respiratory virus test documented in the PED are shown as vertical lines in matte dark red. The vertical dashed lines indicate the statutory COVID-19 lockdown period in Bavaria.

**Figure S5. Shift-level pediatric emergency department visit counts over time, 2020-2024.**

The figure shows the number of PED visits assigned to each nursing shift over calendar time. Blue vertical bars indicate the absolute number of visits per nursing shift. Days with at least 1 positive respiratory virus test documented in the PED are shown as vertical lines in matte dark red. The vertical dashed lines indicate the statutory COVID-19 lockdown period in Bavaria.

**Figure S6. Pairwise shift-level relationships among candidate overcrowding parameters in the pediatric emergency department, 2020-2024.** Pairwise relationships are shown for shift-level overcrowding parameters during the period with available nursing-staffing data. Each point in the lower panels represents one nursing shift. Parameters included the number of visits ending in left without being seen (LWBS) per shift, median patient-to-staff ratio (ps-ratio) per shift, median time to triage per shift in minutes, and median length of stay (LOS) per shift in minutes. Kernel-density distributions are shown on the diagonal, bivariate scatterplots in the lower panels, and Pearson correlation coefficients ($r$) and Spearman rank correlation coefficients ($\rho$) are shown in the upper panels.

**Figure S7. Pairwise day-level relationships among candidate overcrowding parameters in the sensitivity analysis without excluding outliers, 2020-2024.** Pairwise relationships are shown for day-level overcrowding parameters (n = 1,827 days) in the sensitivity dataset before outlier exclusion but after removal of records with non-recoverable missingness or structural implausibility. Parameters included the daily number of visits ending in left without being seen (LWBS), daily median patient-to-staff ratio (ps-ratio; number of children and adolescents simultaneously present in the PED divided by the number of nurses on duty), daily median time to triage in minutes, and daily median length of stay (LOS) in minutes. Kernel-density distributions are shown on the diagonal, bivariate scatterplots in the lower panels, and Pearson correlation coefficients ($r$) and Spearman rank correlation coefficients ($\rho$) in the upper panels.

**Table S1. Distribution of Emergency Severity Index (ESI) levels across nursing shifts, presented as number and percentage of visits, n (%).**

| **Characteristic** | **Early shift** | **Late shift** | **Night shift** | **P-value** |
| --- | --- | --- | --- | --- |
| **ESI** |  |  |  | 1 |
| 1 | 184 (0.40) | 270 (0.41) | 133 (0.46) |  |
| 2 | 4201 (9.19) | 6862 (10.37) | 2949 (10.13) |  |
| 3 | 10511 (22.98) | 13828 (20.90) | 6130 (21.06) |  |
| 4 | 26452 (57.84) | 39604 (59.85) | 15890 (54.60) |  |
| 5 | 4388 (9.59) | 5606 (8.47) | 4000 (13.74) |  |

**Table S2 Comparison of results of the primary ROC analysis with those of the sensitivity analysis (without excluding outliers)**

|  | **Primary analysis (raw)** | | **Sensitivity analysis (raw)** | |
| --- | --- | --- | --- | --- |
| Reference | LWBS $\geq$ 1 | LWBS $\geq$ 2 | LWBS $\geq$ 1 | LWBS $\geq$ 2 |
| AUC | 0.73 | 0.73 | 0.73 | 0.73 |
| Optimal cut-off (Youden index) | 2.15 | 2.21 | 2.21 | 2.21 |
| Sensitivity | 0.69 | 0.76 | 0.74 | 0.63 |
| Specificity | 0.66 | 0.58 | 0.82 | 0.53 |

**Table S3 Comparison of results of the primary time series analysis with those of the sensitivity analysis (without excluding outliers)**

| **Subject of investigation** | **Statistic** | **Primary**  **analysis** | **Sensitivity analysis** |
| --- | --- | --- | --- |
| Nonlinear temporal trend in ps-ratio over the study period | EDF (p-value) | 6.3  (p < 0.001) | 6.5  (p < 0.001) |
| Mean difference in ps-ratio while COVID-19 lockdown period | Level shift | -0.92 | -1.15 |
| Dependence of the ps-ratio on the previous day | Autocorrelation | 0.32 | 0.37 |

Results from generalized additive mixed models with an AR(1) autoregressive correlation structure, penalized regression splines for time across the study period, and adjustment for the COVID-19 lockdown period. EDF denotes the effective degrees of freedom of the nonlinear time trend, representing how flexibly the model captured changes in ps-ratio over the observation period. Higher EDF values indicate greater flexibility of the modelled temporal trend. The results of the primary analysis are compared with those of the sensitivity analysis performed without excluding outliers.
